# Risk factor of *plasmodium knowlesi* infection in Sabah Borneo Malaysia, 2020: A population-based case-control study

**DOI:** 10.1371/journal.pone.0257104

**Published:** 2021-09-10

**Authors:** Abraham Zefong Chin, Richard Avoi, Azman Atil, Khamisah Awang Lukman, Syed Sharizman Syed Abdul Rahim, Mohd Yusof Ibrahim, Kamruddin Ahmed, Mohammad Saffree Jeffree

**Affiliations:** 1 Faculty of Medicine and Health Sciences, Department of Public Health Medicine, Universiti Malaysia Sabah, Kota Kinabalu, Sabah, Malaysia; 2 Faculty of Medicine, Department of Community Health, Universiti Kebangsaan Malaysia Medical Centre, Jalan Yaacob Latiff, Bandar Tun Razak, Cheras, Kuala Lumpur, Malaysia; 3 Borneo Medical and Health Research Centre, Universiti Malaysia Sabah, Kota Kinabalu, Sabah, Malaysia; 4 Faculty of Medicine and Health Sciences, Department of Pathology and Microbiology, Universiti Malaysia Sabah, Kota Kinabalu, Sabah, Malaysia; Instituto Rene Rachou, BRAZIL

## Abstract

**Background:**

In the Malaysian state of Sabah, *P*. *knowlesi* notifications increased from 2% (59/2,741) of total malaria notifications in 2004 to 98% (2030/2,078) in 2017. There was a gap regarding *P*. *knowlesi* acquisition risk factors related to practice specifically in working age group. The main objective of this study was to identify the risk factors for acquiring *P*. *knowlesi* infection in Sabah among the working age group.

**Methods and methods:**

This retrospective population-based case-control study was conducted in Ranau district to assess sociodemographic, behavioural and medical history risk factors using a pretested questionnaire. The data were entered and analyzed using IBM SPSS version 23. Bivariate analysis was conducted using binary logistic regression whereas multivariate analysis was conducted using multivariable logistic regression. We set a statistical significance at *p*-value less than or equal to 0.05.

**Results:**

A total of 266 cases and 532 controls were included in the study. Male gender (AOR = 2.71; 95% CI: 1.63–4.50), spending overnight in forest (AOR = 1.92; 95% CI: 1.20–3.06), not using mosquito repellent (AOR = 2.49; 95% CI: 1.36–4.56) and history of previous malaria infection (AOR = 49.34; 95% CI: 39.09–78.32) were found to be independent predictors of *P*. *knowlesi* infection.

**Conclusions:**

This study showed the need to strengthen the strategies in preventing and controlling *P*. *knowlesi* infection specifically in changing the practice of spending overnight in forest and increasing the usage of personal mosquito repellent.

## Introduction

In the latest World Malaria Report 2020, an estimated 229 million malaria cases occurred worldwide in 2019 compared to 238 million patients in 2010 [1]. Although there were an estimated 19 million fewer malaria cases in 2019 than in 2010, data for 2015–2019 highlighted that no significant progress in reducing global malaria cases with incidence declined by less than 2%. Most of the patients were in the WHO African Region. Malaysia had no cases of human malaria in 2018 and 2019 [1].

As part of its country-owned and nationally-funded malaria strategy, Malaysia has committed to eliminating indigenous human malaria transmission by 2020. In 2017, Malaysia reported a total of 500 cases (local and imported) of the human type of malaria, down substantially from 6141 cases in 2010 [2]. Overall, malaria transmission in Malaysia is mainly in Sabah and Sarawak, two states located on the island of Borneo, where a significant proportion of the population is at risk of the disease. *P*. *knowlesi* malaria, a parasite typically found in monkeys, now accounts for most local cases since its significant discovery in 2004. *P*. *knowlesi*, to date, remains a zoonotic disease without documented sustained human-to-human transmission. In the state of Sabah, *P*. *knowlesi* notifications increased from 2% (59/2,741) of total malaria notifications in 2004 to 98% (2030/2,078) in 2017 [3]. *P*. *knowlesi* cases are increasing over the years and can cause severe disease and death [4]. Increased detection capacity had contributed to the increase in *P*. *knowlesi* prevalence.

Literature has shown several risk factors over the years [5–20]. However, there is a gap regarding *P*. *knowlesi* acquisition risk factors related to practice specifically in working age group. Targeted interventions had varying degrees of success, including insecticide-treated clothing and hammocks (for use in the forest), toxic mosquito baits, and personal insect repellents [21]. The main objective of this study was to identify the risk factors for acquiring *P*. *knowlesi* infection in Sabah among the working age group.

## Methods and materials

### Study design

This was a retrospective population-based case-control study which aim was to assess risk factors of a particular disease using odds ratio. The study site, Ranau district had high incidence of *Plasmodium knowlesi* Malaria in Sabah for the past 6 years. The ratio of case to control was 1:2. Odds ratio (OR) of acquiring *P*. *knowlesi* malaria in exposed subjects relative to unexposed subjects based on occupation factor is 2 [13]. With two controls per case, assuming the probability of exposure among controls (P2) is 0.1, a minimum of 223 *P*. *knowlesi* cases was needed to study to be able to reject the null hypothesis that there is no difference between cases and controls (ie, odds ratio = 1), with 80% power and an α level of 0.05. Factoring in the 10% exclusion rate, the minimum number of cases was 248 cases. The ratio of case to control is 1:2, the minimum number of participants (both cases and controls included) was 744. In this study, we recruited 266 cases, and 532 controls from August 2020 until March 2021.

### Inclusion and exclusion criteria

Cases inclusion criteria were age ≥18 years old; positive microscopy and PCR for *Plasmodium knowlesi* infection; and appropriate informed consent obtained. Cases exclusion criteria were co-infection with other malaria parasites; and travelers from other areas outside the study area. Control inclusion criteria were age ≥18 years old; appropriate informed consent obtained; was investigated as community contact and have negative microscopy based on mass blood smear surveillance activity. Control exclusion criteria were previously recruited as a case which means previous diagnosis of *P*. *knowlesi*; and member of a once selected control household. *Plasmodium knowlesi* cases in Sabah are confirmed by PCR and quality controlled, and cases will be registered under Vekpro system (an open access system provided by Vector-Borne Disease Sector, Ministry of Health Malaysia). Cases of confirmed *Plasmodium knowlesi* malaria over 18 years of age at the time of diagnosis from Ranau district hospital in 2018 and 2019were recruited from the list downloaded from Vekpro system. For selection of control, the selection was made firstly by identifying the village or locality of positive cases. Subsequently, all other houses in the same village or locality except the house of cases will be identified. Individuals primarily residing within the selected house in the previous three weeks during the mass blood smear surveillance activity were numbered and then randomized with a number generator. All households within the village and all individuals within that household had an equal chance of being selected.

### Statistical analysis

Statistical analysis was performed using IBM SPSS software version 23.0 (SPSS, Chicago, IL, USA). Raw data were cleaned and recoded according to produce two categorical data sets for logistic regression analysis (Table 1). The extent of missing data was assessed, and the sensitivity of results to alternative approaches to imputing missing data was explored, wherever necessary. Univariate analysis was conducted using central tendencies and dispersion measures according to the normality of data (mean and standard deviation for normally distributed data, median and interquartile range for non-normally distributed data), and percentages for categorical data. Bivariate analysis for the quantitative stage was conducted using binary logistic regression to compare a categorical independent variable with the dependent variable. Variables correlating with a *p*-value of less than 0.25 and clinically important variables were selected for subsequent multivariate analysis. Multivariate analysis was conducted using multivariable logistic regressions with the statistical significance set at a *p*-value less than 0.05. Hosmer-Lemeshow Test (*p*>0.05) and Classification Table (>70%) were used to measure the performance of the models.

**Table 1:** Coding of variables.

Variable Name	Type	Values and coding
**Pk_positive**	Numeric (binary)	0 = No 1 = Yes
**Age_group**	Numeric (binary)	1 = ≥ 45 years old 1 = < 45 years old
**Gender**	Numeric (binary)	0 = No 1 = Yes
**Use_LLIN**	Numeric (binary)	0 = Yes 1 = No
**Use_repellent**	Numeric (binary)	0 = Yes 1 = No
**Ethnicity**	Numeric (binary)	0 = Others 1 = Native Sabahan
**Occupation**	Numeric (binary)	0 = Non-forest related 1 = Forest-related
**Mal_previous**	Numeric (binary)	0 = Yes 1 = No
**Contact_macaque**	Numeric (binary)	0 = No 1 = Yes
**Income**	Numeric (binary)	0 = Regular 1 = Not regular
**Overnight_forest**	Numeric (binary)	0 = No 1 = Yes
**Telecommunication_home**	Numeric (binary)	0 = Yes 1 = No
**Macaque_pet**	Numeric (binary)	0 = No 1 = Yes
**Marital_status**	Numeric (binary)	0 = Currently married 1 = Others
**Malaria_message**	Numeric (binary)	0 = Previously heard 1 = Never hear
**Forest_dusk**	Numeric (binary)	0 = No 1 = Yes
**Educational_status**	Numeric (binary)	0 = Literate 1 = Illiterate

### Ethics approval and consent to participate

The ethical study clearance was obtained from the Medical Research and Ethics Committee, Ministry of Health Malaysia (Study ID: NMRR-19-3098-51475), and Universiti Malaysia Sabah (Approval Code: JKEtika 3/9 (16). Written consent was obtained from the respondents. The confidentiality of each patient was maintained as no identifiable individual information was recorded.

## Results

The mean age for cases was 40.4 (standard deviation [SD] 14.72) years, and mean of 37.9 (SD 13.04) years for the control group. The median household income for cases was RM900 (interquartile range [IQR] RM300) and RM870 (IQR RM500) for the control group. Based on Table 2, males constituted 214 (80.5%) of the cases and 322 (60.5%) of controls. In both cases and controls, native Sabahan (>90%) dominated the study population. The majority of the population were literate (>95%), having completed minimum primary education than those without formal education. Three out of four cases were currently married, n = 216 (81.2%), while more than two-thirds of control were currently married, n = 386 (72.6%). The majority of the study population were regular income earners (>80% for both groups). Around four out of five of the study population had forest-related occupation, n = 230 (86.5%) for case and n = 405 (76.1%). More than two-thirds of the study population had families who own empty land. The majority of the study population had telecommunication signals at home, n = 234 (88.0%) for cases, and n = 515 (96.8%) for control. More than 90% of the study population heard malaria health messages previously, n = 260 (97.7%) for cases, and n = 484 (91.0%) for control. More than 60% of the study population used LLIN, n = 165 (case), n = 335 (control). Although nearly 80% of cases used mosquito repellant, n = 212, the percentage was lower compared to 87% of control, n = 463. About 48.1% of cases (n = 128) reported spending overnight in the forest compared to only 26.3% of control (n = 140). A bigger proportion of cases reported going into the forest during dusk hours, n = 185 (69.5%) than 48.1% of control (n = 256). A bigger proportion of cases reported contact with macaque n = 160 (60.2%) compared to 45.9% (n = 244) of control. Less than 10% of study population had pet macaque, n = 20 (7.5%) for cases, and n = 18 (3.4%) for control. A total of 84.2% of cases had a history of previous malaria infection, n = 224 compared to only 10% n = 53 in the control group.

**Table 2:** Characteristics of study respondents, summary of bivariate analysis and selection of variables for multivariate analysis.

Variables	Cases N = 266 n (%)	Controls N = 532 n (%)	Crude OR (95% CI)	*p*
**Age**				0.08*
**≥45 years old**	97 (36.5)	162 (30.5)	1.31 (0.96–1.79)	
<45 years old	169 (63.5)	370 (69.5)	1.00	
**Gender**				<0.001*
**Male**	214 (80.5)	322 (60.5)	2.68 (1.89–3.81)	
Female	52 (19.5)	210 (39.5)	1.00	
**Regular income**				0.061*
**No**	35 (13.2)	98 (18.4)	0.67 (0.44–1.02)	
Yes	231 (86.8)	434 (81.6)	1.00	
**Ethnicity**				0.569
**Native Sabahan**	244 (91.7)	494 (92.9)	0.853 (0.464–1.474)	
Others	22 (8.3)	38 (7.1)	1.00	
**Marital status**				0.008*
**Others**	50 (18.8)	146 (27.4)	0.612 (0.426–0.879)	
Currently married	216 (81.2)	386 (72.6)	1.00	
**Educational status**				0.610
**Illiterate**	12 (4.5)	20 (3.8)	1.21 (0.58–2.51)	
Literate	254 (95.5)	512 (96.2)	1.00	
**Occupation**				0.001*
**Forest-related**	230 (86.5)	405 (76.1)	2.003 (1.338–3.000)	
Non-forest related	36 (13.5)	127 (23.9)	1.00	
**Telecommunication signal at home**				<0.001*
**No**	32 (12.0)	17 (3.2)	4.14 (2.26–7.61)	
Yes	234 (88.0)	515 (96.8)	1.00	
**Going to the forest during dusk hours**				<0.001*
**Yes**	185 (69.5)	256 (48.1)	2.46 (1.80–3.36)	
No	81 (30.5)	276 (51.9)	1.00	
**Overnight in forest**				<0.001*
**Yes**	128 (48.1)	140 (26.3)	2.60 (1.91–3.54)	
No	138 (51.9)	392 (73.7)	1.00	
**Having macaque as pet**				0.012*
**Yes**	20 (7.5)	18 (3.4)	2.32 (1.21–4.47)	
No	246 (92.5)	514 (96.6)	1.00	
**Contact with macaque**				<0.001*
**Yes**	160 (60.2)	244 (45.9)	1.78 (1.32–2.40)	
No	106 (39.8)	288 (54.1)	1.00	
**Family own empty land**				0.040*
**Yes**	219 (82.3)	404 (75.9)	1.48 (1.02–2.14)	
No	47 (17.7)	128 (24.1)	1.00	
**Use of LLIN**				0.796
**No**	101 (38.0)	197 (37.0)	1.04 (0.77–1.41)	
Yes	165 (62.0)	335 (63.0)	1.00	
**Use of repellent**				0.007*
**No**	54 (20.3)	69 (13.0)	1.71 (1.16–2.53)	
Yes	212 (79.7)	463 (87.0)	1.00	
**Previous malaria infection**				<0.001*
**Yes**	224 (84.2)	53 (10.0)	48.20 (31.20–74.46)	
No	42 (15.8)	479 (90.0)	1.00	
**Heard/seen malaria health message**				0.001*
**No**	6 (2.3)	48 (9.0)	0.23 (0.10–0.55)	
Yes	260 (97.7)	484 (91.0)	1.00	

The bivariate analysis revealed that male gender, not currently married, having a forest-related occupation, no telecommunication signal at home, going to the forest during dusk hours, spending overnight in the forest, having pet macaque, having contact with a macaque, the family owning empty land, not using mosquito repellent, and history of previous malaria infection, and never heard or seen malaria health message were found to have a crude association with *P*. *knowlesi* infection. Variables with a p-value of ≤0.25 and other clinically important variables were included in multivariate logistic regression analysis (marked with *) (Table 2). Forward and backward logistic regression methods applied. The odd ratio (β) for the significant factors shows the increase (or decrease if the ratio is less than one) in odds of being in one outcome category (positive or negative for Pk infection) when the value of the predictor increases by one unit. From Table 3, individuals who were male gender were 2.71 times more likely than individuals who were female gender to acquire *P*. *knowlesi* infection, all other factors being equal (AOR = 2.71; 95% CI: 1.63–4.50). Individuals who practiced spending overnight in the forest were 1.92 times more likely than individuals who do not practice spending overnight in the forest to acquire *P*. *knowlesi* infection, all other factors being equal (AOR = 1.92; 95% CI: 1.20–3.06). Individuals who did not use mosquito repellent were 2.49 times more likely than individuals who used mosquito repellent to acquire *P*. *knowlesi* infection, all other factors being equal (AOR = 2.49; 95% CI: 1.36–4.56). Individuals with a history of previous malaria infection were 49.34 times more likely than individuals without a history of prior malaria infection to acquire *P*. *knowlesi* infection, all other factors being equal (AOR = 49.34; 95%CI: 39.09–78.32). The model was fitted with the Nagelkerke R Square = 0.634 and Hosmer–Lemeshow test (*p* = 0.333), with the overall correctly classified percentage of 88.1% (>70%).

**Table 3:** Summary of multivariable logistic regression analysis of risk factors for acquiring *P*. *knowlesi* infection.

Variables	Adjusted OR (95% CI)	*p*
**Gender**		
**Male**	2.71 (1.63–4.50)	0.000
**Female**	1.00	
**Overnight in forest**		0.006
**Yes**	1.92 (1.20–3.06)	
**No**	1.00	
**Use of repellent**		0.003
**No**	2.49 (1.36–4.56)	
**Yes**	1.00	
**Previous malaria infection**		0.000
**Yes**	49.34 (39.09–78.32)	
**No**	1.00	

## Discussion

Age was a risk factor of *P*. *knowlesi* infection in bivariate analysis, but was not an independent predictor in the final multivariable model. Previous studies showed that all ages were susceptible to infection, with cases also reported in Malaysian children [22], and Vietnamese children [23,24]. Patients with *knowlesi* malaria demonstrated a wide age distribution (median 33, IQR 20–50, range 0.7–89 years). The presence of familial clustering of cases had been demonstrated, indicating transmission is probably now occurring peri-domestically, which may be linked to deforestation or land-use change in these environments [5], or other yet to be determined factors. Another study reported mean age to get *P*. *knowlesi* infection is 44.9 [6]. The scope of this study was 18 years and older because the evidence of peri-domestic infection has not been widely explored and is different than the more extensively studied risk predominantly linked to exposure activities [14]. Respondents with history of previous malaria infection were more likely than those without a history of malaria infection to acquire *P*. *knowlesi* infection as found in this study. This factor was also an independent predictor in the multivariable model. This was most likely due to their activities and practices. Moreover, as of date, there is lack of evidence supporting any form of immunity from previous malaria infections. Current available control measures which were effective against human indigenous malaria have shown little success in controlling *P*. *knowlesi* malaria due to the outdoor biting nature of its vector [25]. A clinical parameter study may be helpful to explore this factor. Many of the respondents (more than 15%) could not give an estimated monthly income because their income was not regular. They supported their daily lives with natural resources and financial assistance from the government. Although a significant percentage had a steady income, many (>80%) still earned less than RM2,000 per family per month, which was below the Poverty Line Income (PLI) per month, put at RM2,537 [26]. Despite this fact, having no regular income was not a statistically significant risk factor for developing *P*. *knowlesi* infection. We may relate income to the ability to procure mosquito repellent, which the Ministry of Health does not provide, Malaysia, under its Vector Borne Diseases Control Program Sector. This factor can be explored in future research.

There was a correlation between income and level of education obtained [27]. In this study, a proportion of illiterate respondents had never attended formal education but was not significantly associated with *P*. *knowlesi* infection. Siri found that educating women could enhance their essential life skills, trust in public health systems, and their ability to become more conscious about the various factors that affect the health of their children [28]. Poor knowledge and understanding of malaria could subsequently lead to adverse attitudes and systems towards malaria prevention [19]. This evidence can be applied to advocate improving the educational level of the local population for better absorption of malaria health message and empowerment of the local community to build resilience in preventing *P*. *knowlesi* infection despite the fact that the variable never hearing malaria health message was not statistically significant to be associated with *P*. *knowlesi* infection. Having an internet signal at home is an essential factor in ensuring the timely conveyance of health messages. Not having an internet signal at home caused many individuals to enter the forest at night for a good internet signal, which led to them being infected with *P*. *knowlesi*. Although there was a statistically significant association between not having a telecommunication signal at home with *P*. *knowlesi* infection in the bivariate analysis, it was not an independent predictor in the multivariable model.

The male gender presented the more significant proportion of the two genders in both groups of control and cases. This factor was a statistically significant factor for *P*. *knowlesi* in bivariate analysis and was also an independent predictor in multivariable analysis. This confirmed the findings reported in a previous study, which stated that men had higher risks of *P*. *knowlesi* exposure, especially adult men working in agricultural areas, had the highest risk of *P*. *knowlesi* infection [13]. Another study conducted in Aceh also confirms similar findings [14]. As this study was conducted in Ranau District in Sabah, with more than 90% of its population being Native Sabahan, it was not surprising that being of Native Sabahan ethnicity was not a statistically significant factor for *P*. *knowlesi* infection. We believe that many of Native Sabahan in this study acquired *P*. *knowlesi* infection due to their respective residential area or occupational group. The poor were concentrated in Bumiputera, with a higher incidence of poverty, especially in Sabah, where the poor were concentrated in rural areas where *P*. *knowlesi* incidence is high [26].

Changes in land use such as shifts in agricultural practices, deforestation, and forest fragmentation have been proposed as key drivers in the emergence of *P*. *knowlesi* infection [29]. This complex interaction could be related to the proximity of vectors and include conflict around the availability of food for macaques from human agricultural practices such as cultivated fruit orchards, rice paddies, or cornfields, in addition to traditional or changing techniques such as trapping or hunting monkeys for pets or food. Despite spending most of their time in the forest, macaques invade farmland in search of food. The percentage of the total macaque population in daily contact with the farm is unknown. In order to understand more on *P*. *knowlesi* transmission, we require timely information on degree of changing forest cover and land use and the efects on the distribution of vectors and hosts of *P*. *knowlesi* and parasite transmission rate through increased in surveillance activities utilising latest technology [30]. Vythilingam et al. found that the prevalence of *P*. *knowlesi* infection of macaque in the wild (forests) was exceptionally high at 97%., compared to urban macaques, which were infection-free [31]. Lee et al., also found a prevalence of 87% in Sarawak among long-tailed macaques [32]. Many studies have described the association of *P*. *knowlesi* with occupation. Occupations such as forestry, agriculture, and hunting had been identified as risk factors for getting *P*. *knowlesi* infection [7,13]. A prospective study of malaria patients from a referral catchment area of north-western Sabah described 92% (119/130) of PCR confirmed *P*. *knowlesi* malaria cases having forest or plantation exposure, including living within a 20 minute of unclassified forest type or a plantation (*p* = 0.001 and 0.015, respectively) [9]. Apart from occupation, some reports of *P*. *knowlesi* infection from traveling activity in a popular low-risk travel destination [33]. Ranau district, a frequent travel place for local and international tourists alike, had this risk to consider among travelers. *An*. *Balabacensis*, the vector implicated in Sabah for *P*. *knowlesi*, prefers to breed in-ground pools formed in fruit orchard, rubber, and palm oil plantations [34]. With this precedence, there was no surprise that both having forest-related occupation and contact with macaque were statistically significant risk factors for *P*. *knowlesi* infection in this study, albeit not being an independent predictor in the multivariable model. With human populations increasingly encroaching on and supplanting macaque habitats, there is intense selective pressure for the parasite to switch its natural mammalian host. Human-to-human transmission of *P*. *knowlesi* has been shown to occur under experimental conditions [35]. A study in Singapore showed more than 80% of wild macaques sampled were infected with *Plasmodium* parasites [36]. Keeping all these in mind, there is an urgent need for risk mapping of macaque density as well as surveillance of wild macaque.

*P*. *knowlesi* infection reported in two clusters in Aceh, Indonesia, revealed a history of spending overnight in the forest, with or without protection, in the area where macaques were abundant [10]. A local study found a strong association between traveling in the forest area at night [13]. Individuals with a workplace location in or near the forest and requiring overnight stays and individuals who visited the forest in the previous month for any reason had higher odds of *P*. *knowlesi* infection compared to those who don’t [14].

Vector mosquitoes, members of the *An*. *leucosphyrus* group are found throughout forested regions of Malaysia and are particularly associated with dense jungle and forest fringes [37]. *An*. *leucosphyrus* mosquitoes are exophagic, typically feeding and resting outdoors after dusk. To this extent, exploration was also conducted to find an association between spending overnight in the forest and going to the forest during dusk hours. Although both these factors were statistically significant during bivariate analysis, only spending overnight in forest was an independent predictor in the multivariable model. Further study needs to be conducted to determine the duration of time spent in forests with forest-related occupation or activities to produce a more reliable model that fits both variables.

The *Leucosphyrus* mosquito complex has been predicted to occur in areas with high forest cover but no intact forest cover [34]. The sparse data and low volume of data for this complex mean that these predictions should be interpreted cautiously. This complex is responsible for *P*. *knowlesi* in the region in which *knowlesi* malaria occurs most frequently in Borneo of Malaysia. Deforestation (loss of intact forests) occurs [11,12]. The conversion of intact forest to disturbed forest and the resulting impact on the likelihood of members of the *Leucosphyrus* complex could be a factor in to increase of *P*. *knowlesi* cases. A study in the northern part of Sabah in Borneo, Malaysia recently found an association between two forest variables, namely forest loss and total cover within two km of a village, and the estimated incidence of *knowlesi* malaria at the village level in the two districts studied, but this does not differentiate between intact and disturbed forest [38]. In our study, there were individuals with family empty land opening activities infected with *P*. *knowlesi*. Although the bivariate analysis stage of this study elicited the association between family owning empty land and *P*. *knowlesi* infection, it was not an independent predictor in the multivariable model.

In Malaysian Borneo, studies found that M. fascicularis populations were declining due to logging activities. Still, their local abundance was higher in areas logged ten years previously than in unlogged forests [39]. M. fascicularis populations can occupy a wide range of habitats. More importantly, the distribution of this species encompasses many locations close to human habitation (urban areas) or activity (disturbed forests, orchards, croplands, etc.) [40]. Apart from that, there is evidence of zoonotic transmission of diseases harbored by companion animals about the close contact with their owners. There is evidence that *Macaca fascicularis* and *M*. *nemestrina* monkeys are kept as pets on Sulawesi [41]. The same practice was found in Ranau District, whereby investigation of a suspected peri-domestic *knowlesi* malaria infection of a young child has led to the discovery of the practice of having macaque as a pet which was subsequently tested and was found to have *P*. *knowlesi* parasite (unpublished). However, in this study, although there was a statistically significant association between having macaque as a pet and *P*. *knowlesi* infection, it was not an independent predictor in the multivariable model.

With malaria elimination at the grasp of the hands for Malaysia, one of the main challenges is the prevention and control of zoonotic *knowlesi* malaria in rural areas where there is a large pool of parasites in the macaque reservoir hosts. A reduction in these macaque reservoir hosts would be one option, but implementing such a measure would be impractical and burdensome. The most common vector control strategies for malaria are Long Lasting Insecticidal Nets (LLINs) and the Indoor Residual Spraying (IRS) of houses; both focused on protecting against human malaria. The mosquitoes’ outdoor biting and resting behaviour hinder the effectiveness of these methods due to the zoonotic reservoirs in the macaques. Preventing humans from being bitten by vectors would be the primary prevention and control of *knowlesi* malaria in these settings. It would involve the use of insect repellents. Still, these are prohibitively expensive for subsistence farmers, hunters, logging camp workers, and other rural people whose daily activities take them to the forest and forest fringe. The use of insecticide-impregnated hammocks had been successful in controlling forest malaria in Vietnam [42]. Although some people stay overnight in the forest of Malaysian Borneo, hammocks were not traditionally used here. So much a control measure would be challenging to implement successfully.

In this study, non-users of LLIN were not shown to have higher odds than users to acquire *P*. *knowlesi* infection. Non-users of mosquito repellant, on the other hand, were at higher odds than users of mosquito repellant to develop *P*. *knowlesi* infection and was an independent predictor in the multivariable model. Insecticide-treated clothings (ITC) was reported as effective against bites from *Anopheles* mosquitoes [43,44]. However, ITC is not easily accessible, thus was not assessed in this study [45]. Further research is warranted to explore the potential of ITC for reducing zoonotic and human malaria, particularly regarding its scalability, cost-effectiveness, duration of durability, safety under variable environmental working conditions, resistance to UV light exposure, and washing [45–47].

The possibility of eliminating *knowlesi* malaria is limited because forests that harbor *Anopheles* mosquitoes and macaque monkeys will remain a reservoir for the zoonotic transmission of *P*. *knowlesi*. The risk for *P*. *knowlesi* infection in humans is highly variable. There is a sense of urgency to be on top of controlling and preventing *Pk* infection. The problem is compounded further because there are numerous vector species with highly variable anthropophilic and many simian host species [48,49]. Increasing knowledge on mosquito bite prevention has been shown to reduce *Pk* incidence effectively [50].

### Limitation of study

The most frequently cited disadvantage in case-control studies is the potential for recall bias. Recall bias in case-control studies is that those with positive results are more likely to remember and report exposures. Keep in mind that people with this illness are more likely to think more about these exposures than healthy. Case-control studies, usually of retrospective nature, can correlate with the outcome, but causation cannot be determined. These studies seek to find a correlation between past events and current conditions.

## Conclusion

In summary, male gender (AOR = 2.71; 95% CI: 1.63–4.50), spending overnight in forest (AOR = 1.92; 95% CI: 1.20–3.06), not using insect repellent (AOR = 2.49; 95% CI: 1.36–4.56) and ever diagnosed with malaria (AOR = 49.34; 95% CI: 39.09–78.32) were found to be independent predictors of *P*. *knowlesi* infection. This study showed the need to strengthen the strategies in preventing and controlling *P*. *knowlesi* infection specifically in changing the practice of spending overnight in forest and increasing the usage of personal mosquito repellent.

### Key recommendations

Many more factors can be explored more profoundly, such as the effect of duration in forest and information on the risk of infection in the current online database, Vekpro, as guided by the latest evidence. Since many of the respondents are living below the poverty line, the inclusion of non-governmental organizations (NGOs) is imperative to increase efforts for prevention of *P*. *knowlesi* infection by providing complimentary assistance in terms of mosquito bite prevention equipment, on top of the aid given by the Sabah State Health Department. The population of this study cannot be separated from entering the forest for various reasons, mainly for livelihood, despite much evidence pointing towards the increased risk of getting *P*. *knowlesi* infection. Thus, there is a need to increase health promotion efforts to empower the local community and build resilience to prevent a malaria infection and improve livelihood in the same setting.

## Abbreviations

*Pk*: *Plasmodium knowlesi*
PCR: Polymerase Chain Reaction
AOR: Adjusted Odds Ratio
LLIN: Long Lasting Insecticide Treated Net
IRS: Indoor Residual Spraying
OR: Odds Ratio
GIS: Geographic Information System
CI: Confidence Interval
COR: Crude Odds Ratio
ITC: Insecticide-treated clothings
